# The repair of full-thickness articular cartilage defect using intra-articular administration of *N*-acetyl-d-glucosamine in the rabbit knee: randomized controlled trial

**DOI:** 10.1186/s12938-015-0100-y

**Published:** 2015-11-18

**Authors:** Nai-Jen Chang, Yi-Ting Lin, Chih-Chan Lin, Hsueh-Chun Wang, Horng-Chaung Hsu, Ming-Long Yeh

**Affiliations:** Department of Sports Medicine, Kaohsiung Medical University, Kaohsiung, Taiwan; Department of Biomedical Engineering, National Cheng Kung University, 1 University Rd., Tainan, 701 Taiwan; Laboratory Animal Center, Department of Medical Research, Chi-Mei Medical Center, Tainan, Taiwan; Department of Orthopedics, China Medical University Hospital, Taichung, Taiwan; Medical Device Innovation Center, National Cheng Kung University, Tainan, 701 Taiwan

**Keywords:** Glucosamine, Intra-articular injection, Tissue engineering, Cartilage

## Abstract

**Background:**

Although various alterative models of therapy are used for cartilage repair, no definite conclusion has been reached. Glucosamine (GlcN) is widely used as a nutritional supplement. However, the clinical- evidence-based outcome of GlcN administration remains controversial. *N*-acetyl-d-glucosamine (GlcNAc), a derivative of GlcN, shows chondroprotective activity and mediates the activation of articular chondrocytes. Therefore, we investigated the effect of intra-articular administration of GlcNAc in rabbits’ knee joints with experimental full-thickness articular cartilage (FTAC) defects.

**Methods:**

Twelve male adult New Zealand white rabbits, providing 24 knees, were used in this study. FTAC defects were created in the high-weight-bearing area of the medial femoral condyles of bilateral knees. All rabbits were randomly allocated to analysis at postsurgical week 4 or postsurgical week 12. In the week 4 group, rabbits’ knees (six per group) were intra-articularly injected with normal saline or with GlcNAc twice per week for 3 weeks, beginning 1 week postoperatively. In the week 12 group, the rabbits’ knees (six in each group) were intra-articularly injected with normal saline or with GlcNAc twice per week for 4 weeks, beginning 1 week postoperatively. Rabbits were sacrificed at 4 or 12 weeks after surgery for macroscopic, histological and radiological examinations of the knee joints.

**Results:**

All rabbits had no systemic or local adverse effects. The saline and GlcNAc groups showed visible differences in healing of the FTAC defect at the end of testing. At week 4, the GlcNAc group had a higher level of collagen type II (COL II) and showed up-regulated production of transforming growth factor (TGF)-β2 and TGF-β3, suggesting the involvement of endogenous growth factors. At week 12, the GlcNAc group displayed formation of hyaline-like cartilage regeneration with mature chondrocytes (SOX9+), robust glycosaminoglycan (GAG) content, and positive COL II content in both the adjacent cartilage and reparative sites. However, the saline group demonstrated mainly fibrocartilage scar tissue, indicating COL I expression. Furthermore, the GlcNAc group had significantly higher bone volume per tissue volume and higher trabecular thickness than the saline group.

**Conclusions:**

Intra-articular GlcNAc may promote the repair of experimental FTAC defects in the rabbit knee joint model.

## Background

The repair of articular cartilage defects is particularly challenging because of cartilage’s hypocellularity and insufficient nutrient supply, and the inability of bone marrow stem cells or resident chondroprogenitor cells to form hyaline cartilage. Current clinical therapeutic treatments for cartilage repair include intra-articular therapy, e.g., non-steroidal anti-inflammatory drugs, corticosteroids, dextrose prolotherapy, hyaluronic acid (HA) or platelet-rich plasma [1, 2], microfracture, autologous chondrocyte implantation, and osteochondral transplantation techniques [3, 4]. However, these treatment options, particularly for large cartilage defects, present not only the formation of fibrocartilage scar tissue but also poor integration of hyaline cartilage in the host [5, 6]. Hence, regarding their effectiveness for large cartilage lesions, there is a lack of both clinical consensus and reproducible evidence that currently available treatment significantly changes the progression of osteoarthritis, based on prospective, randomized studies [7–9]. Thus far, no defined conclusion for cartilage repair has been reported.

Glucosamine (GlcN) is one of the most widely utilized dietary supplements. It is also believed to stimulate the metabolism of chondrocytes, thus providing beneficial effects on articular health. However, the clinical outcomes of GlcN administration orally remain controversial due to the various formulations and purity of GlcN [10–13]. GlcN intake as a nutritional supplement is commercially available in one of three forms: GlcN hydrochloride, GlcN sulfate, and *N*-acetyl-d-glucosamine (GlcNAc). Since a molecule of pure GlcN is essentially unstable, it should be salt-stabilized (e.g., chloride or sulfate) [14]. In particular, GlcNAc, classified as a monosaccharide derived from glucose, has a relatively stable structure.

Articular cartilage repair is determined by the growth microenvironment in the synovial joint. Ex vivo, administration of GlcN may modify disease activity, but a higher concentration of GlcN (>10 mM) depresses glucose transport, leading to decreased cell viability, depletion of proteoglycan synthesis, and further damage to the articular cartilage [15]. However, high concentrations of GlcNAc (>50 mM) have no inhibition on proteoglycan synthesis [16]. Previous in vitro studies reported that GlcNAc potentially promotes chondrogenesis of mesenchymal stem cells and enhances HA and glycosaminoglycan (GAG) synthesis [17]. Furthermore, GlcNAc can be hydrolyzed to GlcN both in vivo and in vitro in applications for cartilage regeneration and anti-inflammatory activity [18–23]. Regarding pharmacokinetics, clinically relevant dosing of glucosamine HCl in serum and synovial fluid concentrations that are at least 500 fold lower than those reported to modify chondrocyte anabolic and catabolic activities in tissue and cell culture experiments. Most of the dietary glucosamine on pain and joint space may be secondary to its effects on nonarticular tissues, such as the intestinal lining, liver, or kidney, since these may be exposed to much high levels of glucosamine following ingestion [24]. In other words, GlcN is almost absorbed from the gastrointestinal tract through oral administration, but very little diffuse into articular cartilage [25]. On the other hand, intravenous injection of GlcN within 1 h, approximately 30 percent is excreted in the urine. Some may be excreted in the gut, and the remainder is metabolized in the body [26]. Alternatively, local intra-articular injection with the preferred agent can directly supply an in situ requirement of the growth microenvironment. Moreover, compared to GlcN, GlcNAc has been shown to have different biological activities including uptake and effects on glucose transport, glucose transporter expression, and synthesis of sulfated GAG and HA [27]. A previous study investigated oral GlcNAc and GlcN administration on plasma total free amino acid (PFAA) concentrations in dogs [28]. GlcNAc group has the higher Glu, Gly, and Ala concentrations after 1 h administration compared with GlcN group. The three kind of non essential amino acid are the main components of type II collagen in cartilage.

Therefore, we investigated whether intra-articular injection of GlcNAc could provide a safe niche for the repair of full-thickness articular cartilage (FTAC) defects in rabbit knee joints.

## Methods

### Reagent

GlcNAc (molecular formula: C_8_H_15_NO_6_) was purchased from Sigma (SI-A3286, MO, USA). It was dissolved in 0.9 % saline solution, and sterile filtered The sterilized solution was stored at 4 °C.

### Surgical procedure

All surgical procedures and animal ethics were approved by the Animal Care and Use Committee of National Cheng Kung University. Twelve 4–5-month-old male New Zealand white rabbits (Livestock Research Institute, Taiwan) weighing 2.5–3 kg were used in this experiment, providing 24 knees. Before surgery, anesthesia was induced with Zoletil 50 (25 mg/kg) (Virbac, France) and maintained with a mixture of 2 % isoflurane (Panion & BF Biotech Inc., Taiwan) and oxygen/nitrous oxide (1/0.4 L/min). Both knees of each rabbit were shaved and disinfected with 10 % ethanol-iodine solution. Longitudinal incisions were made along the parapatellar and capsular ligaments. The medial femoral condyle was exposed by lateral patellar dislocation. A critical FTAC defect, 3 mm in diameter and 3 mm deep, was created with an electric drill in the high-weight-bearing region of the medial femoral condyle, simultaneously irrigated with saline for cooling, followed by patellar relocation and joint closure. After surgery, the rabbits were permitted to move freely in their cages. Appetite, body weight, skin wound healing and functional activities were monitored.

### Intra-articular injection

Each animal was given either saline or GlcNAc. At 7 days after surgery, in the week 4 group, rabbits’ knees (six knees per group) were injected with 0.3 ml of normal sterile saline (0.9 % NaCl) or intra-articularly with GlcNAc solution twice per week starting 1 week post-surgery for a period of 3 weeks. In the week 12 group, rabbits’ knees (six knees per group) were injected with normal saline or intra-articularly with GlcNAc twice per week starting 1 week post-surgery for a period of 4 weeks. The single dose of GlcNAc was 80 mg/0.3 ml per joint. In addition, the recommended frequency of intra-articular injection for optimal therapeutic efficacy is twice per week [18].

### Macroscopic evaluation

At the end of testing, the outcome of the FTAC defect was first observed via macroscopic appearance based on the modified Wayne’s grading scores (Table 1) [29]. The neo-tissue coverage, color and surface condition were assessed independently by two investigators. The maximum possible total score was 12 points.

**Table 1:** A modified Wayne’s grading scale scoring system for gross appearance

Macroscopic appearance	Points
Coverage	
>75 % fill	4
50–75 % fill	3
25–50 % fill	2
<25 % fill	1
0 % fill	0
Tissue color	
Normal/whitish	4
>25 % yellow/reddish	3
>50 % yellow/reddish	2
>75 % yellow/reddish	1
100 % yellow/reddish	0
Surface	
Normal, smooth	4
Smooth but raised	3
25–50 % irregularity/fibrillation	2
50–75 % irregularity/fibrillation	1
>75 % irregularity/fibrillation	0
Total	12

### Micro-CT evaluation

Micro-computed tomography (CT) can provide quantitative and qualitative measurements to identify the healing of bone architecture. A high-resolution microtomograph 1076 scanner (SkyScan, Kontich, Belgium) was employed to scan the distal femur FTAC defect in the saline and GlcNAc groups at 4 and 12 weeks after surgery. The voltage and beam current of the X-ray source were set at 50 kV and 160 μA, respectively. The pixel size was set at 18 μm in resolution. The samples were scanned with 360° rotation with 1° of rotation interval.

The SkyScan software package, including SkyScan CT-Analyzer v.1.8, and CT-Volume v.2.0, were used to reconstruct the image data and visualize the representation of the newly formed bone. From the data set, a cylindrical region of interest (ROI) 3 mm in diameter within the repaired site was selected for analysis. This ROI corresponds to the original defect region. The bone volume per tissue volume (BV/TV) and trabecular thickness (Tb.Th) were obtained to evaluate the bone volume density and to measure the thickness of trabecular structures, respectively.

### Histological and immunohistochemistry processing

All histological sections were performed by the Department of Pathology at Chi-Mei Medical Center. The femur ends were dissected, fixed in 10 % neutral buffered formaldehyde solution, decalcified and embedded in paraffin blocks. From each sample, the tissue was sliced by a microtome into 4 μm thick sections for histological staining. Hematoxylin and eosin (H&E) staining was performed to allow general observations; Masson’s trichrome stain was used for total collagen and alignment; Alcian blue staining was performed for GAG synthesis. Furthermore, immunohistochemistry analysis was conducted to confirm the expression of collagen type I (COL I, fibrocartilage), collagen type II (COL II, hyaline cartilage), endogenous growth factors (i.e., TGFβ-2 and TGFβ-3) as well as SOX9 (marker of mature chondrocytes) for the repair of FTAC defects. All staining protocols followed the manufacturers’ recommended guidelines. Briefly, the sections were treated with endogenous peroxidase and incubated overnight with a monoclonal primary antibody diluted 1:100: COL I (Bioworld Technology), COL II (Bioss), TGFβ-2 or TGFβ-3 (Spring Bioscience), or SOX9 (Bioss). The rabbit/mouse HRP-DAB polymer detection kit (BioSB) was used as the secondary antibody at room temperature for 30 min. Then, DAB (3,3′ diaminobenzidine) staining in brown represented positive immunoreactivity. The sections were counterstained with hematoxylin (BioSB) and then recorded using a light microscope (Olympus IX71, Tokyo, Japan) and a digital CCD camera (Olympus DP72, Tokyo, Japan).

### Statistical analysis

The software package SPSS v17.0 was used for the statistical analysis. All of the data (six knees per group at week 4 or 12) are expressed as the mean ± standard error of the mean. Owing to the concern of repeated measurements from bilateral knees on the same individual [30], a linear model using generalized estimating equations (GEE) was employed for statistical comparison at each time point. *P*-values <0.05 were defined as statistically significant. The statistical power of this study was 0.9 as determined by the mean, variation, and sample size.

## Results

### Macroscopic observations and scoring in the FTAC model

**Fig. 1:**
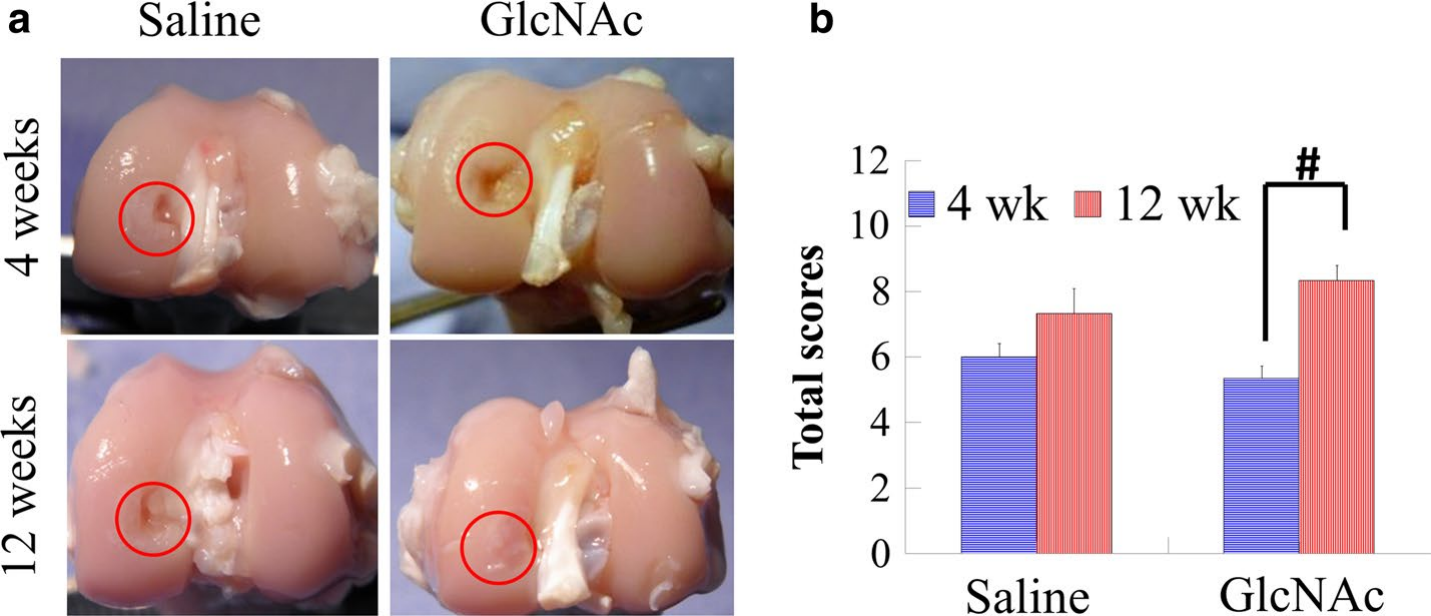
**a** Representative images of the gross appearances at 4 and 12 weeks after operation. *Circles* indicate the repaired osteochondral defect area. **b** Quantitative scores of the gross appearance. ^#^ *p* < 0.01

The gross appearance of FTAC defects was assessed at both 4 and 12 weeks after surgery (Fig. 1a). No infection, synovitis or osteophyte formation was observed at either time point. At week 4, the defect areas in both the saline and GlcNAc groups were visibly concave although new tissue had developed inwards from the outer area of the defect edges. At week 12, the GlcNAc group had formed a transparent neo-cartilage-like tissue. Meanwhile, the saline group showed incomplete filling, with hybrid whitish/yellow tissues and an obvious fissure in the reparative site (Fig. 1a).

The total scores in both saline (6.00 ± 0.40) and GlcNAc groups (7.33 ± 0.76) were comparable at week 4 after surgery. However, at week 12 after surgery, the total score for the GlcNAc group (8.33 ± 0.47) was superior to that for the saline group (5.36 ± 0.37). In addition, the GlcNAc group showed a significant increase in total scores from 4 to 12 weeks (*p* < 0.01) (Fig. 1b).

### Subchondral bone formation using Micro-CT evaluation

**Fig. 2:**
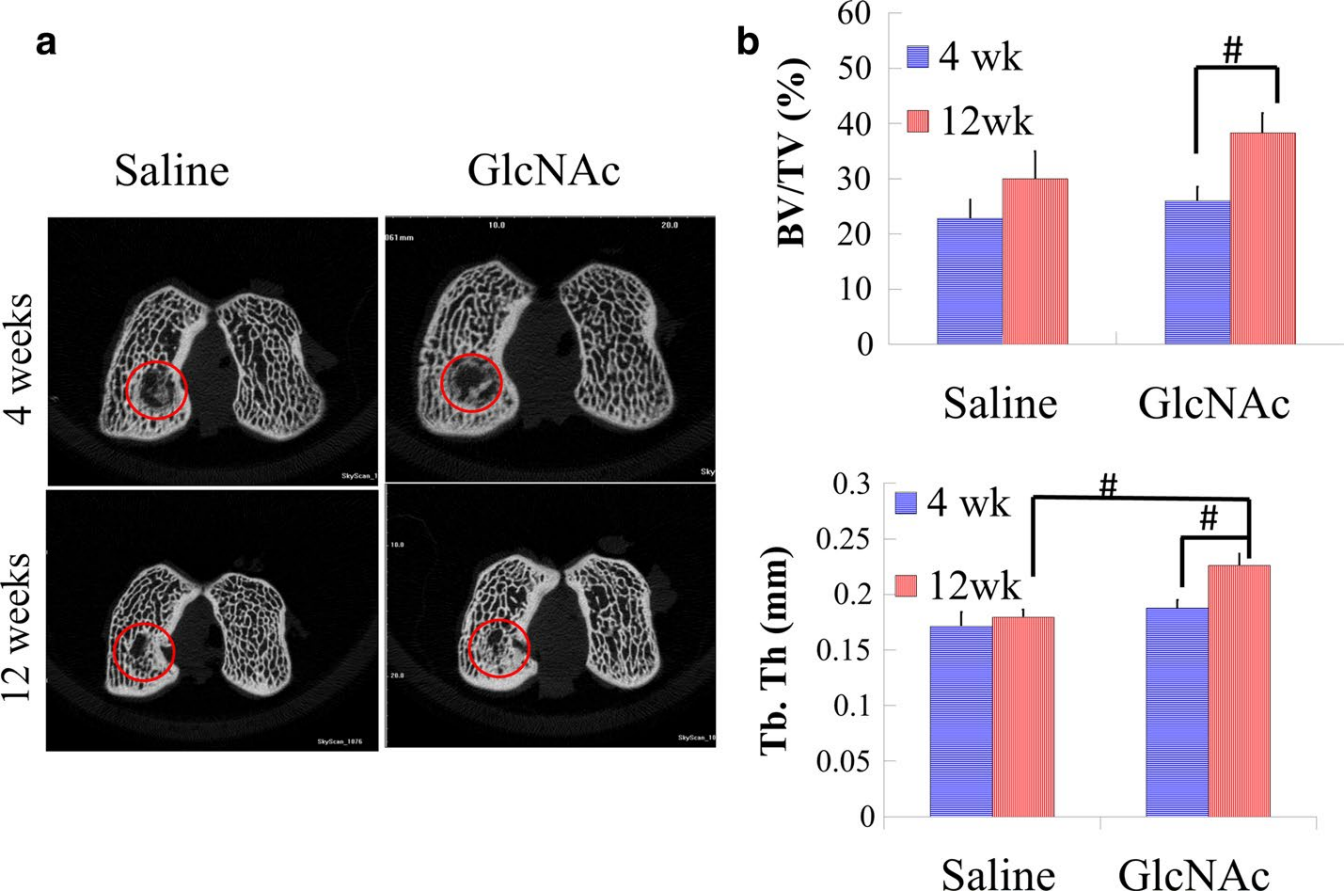
Analysis of bone regeneration. **a** Micro-CT images of bone assessment at 4 weeks and 12 weeks after operation.* Circles* indicate the repaired osteochondral defect area. **b** Quantitative scores of the ratio of bone volume to tissue volume (BV/TV) and of the thickness of trabecular bone (Tb.Th). ^#^ *p* < 0.001

Micro-CT images of medial condyles at weeks 4 and 12 after surgery are shown in Fig. 2a. The repairing pattern of newly mineralized tissue was edge-to-center, indicating development from the outer area of the defect. At week 4, regenerative osseous tissues in saline and GlcNAc groups were comparable. However, at week 12, the GlcNAc group had more newly osseous tissues and integrating trabecular bones than the saline group. In addition, BV/TV and Tb.Th were used as measures of subchondral bone regeneration. With respect to BV/TV, the values in the GlcNAc group (38.3 ± 3.5) were markedly higher than the saline group (30.0 ± 5.0), particularly at week 12 (Fig. 2b). The GlcNAc group had a 1.5-fold greater increase in BV/TV compared to the saline group from week 4 to week 12 (Fig. 2b). Moreover, the GlcNAc group showed a significant increase in BV/TV from week 4 (26.0 ± 2.6) to week 12 (38.3 ± 3.5) (p = 0.002) (Fig. 2b). With respect to Tb.Th, at 12 weeks, the values in the GlcNAc group (0.23 ± 0.01) were significantly higher than those in the saline group (0.19 ± 0.01) (p < 0.001). In addition, the GlcNAc group showed a significant increase in Tb.Th over time (p < 0.001) (Fig. 2b).

### Histological findings

#### At 4 weeks after operation

**Fig. 3:**
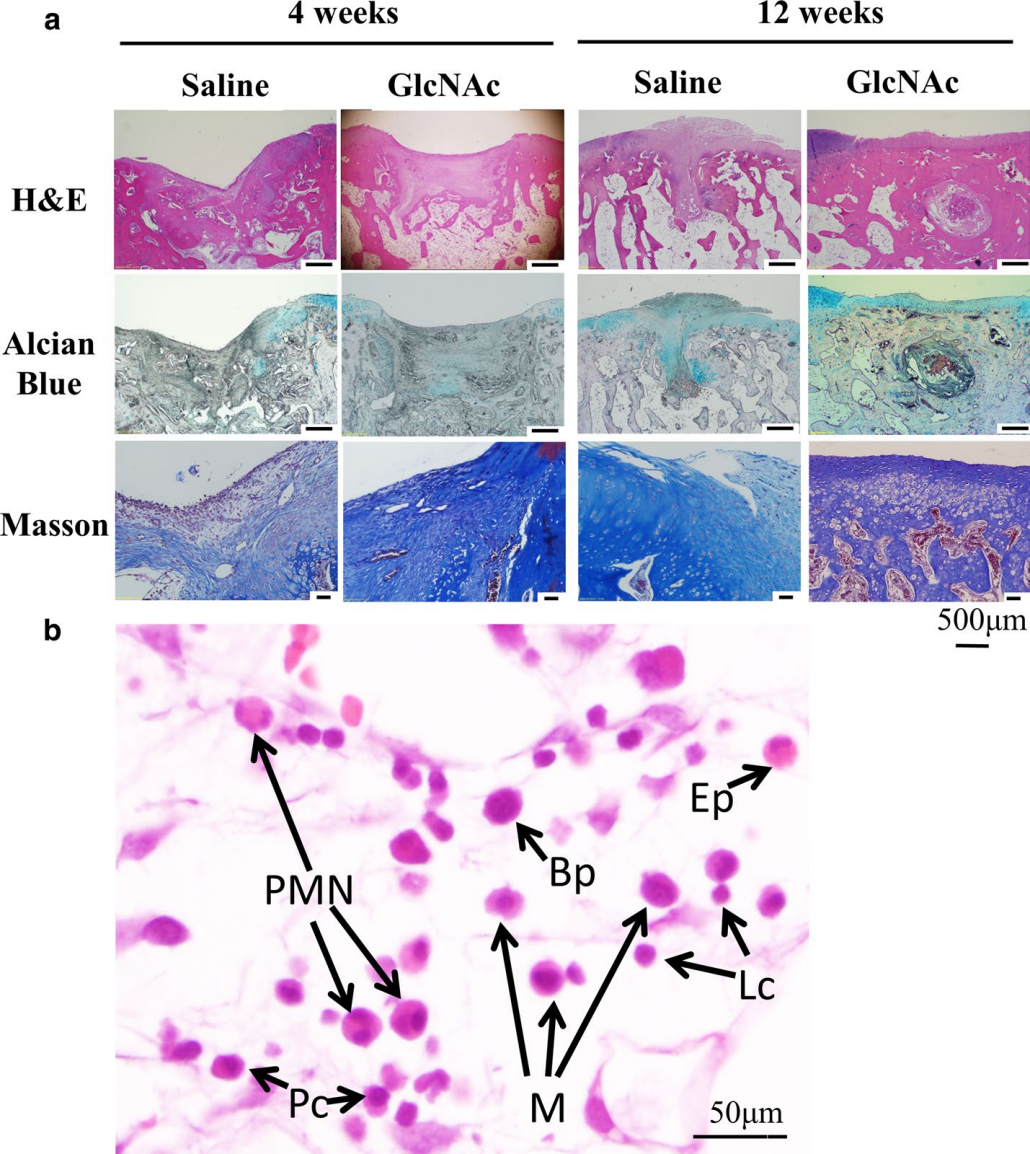
**a** Representative histological images of the repaired area using H&E, Masson’s trichrome, and Alcian blue staining at 4 and 12 weeks after operation. **b** The saline group contained representative inflammatory cells, including plasma cells (Pc), lymphocyte (Lc), polymorphonuclear neutrophils (PMN), basophil (Bp), eosinophil (Ep) and Macrophage (M), in the reparative regions at 4 weeks

In the saline group, the defect regions were filled with hyperplasic blood vessels, contained disoriented fibrous tissue together with fibroblast-like cells, and showed a shortage of GAG synthesis (Fig. 3a). Furthermore, representative inflammatory cells, including plasma cells, lymphocytes and polymorphonuclear neutrophils (PMNs) were found in the reparative region (Fig. 3b).

**Fig. 4:**
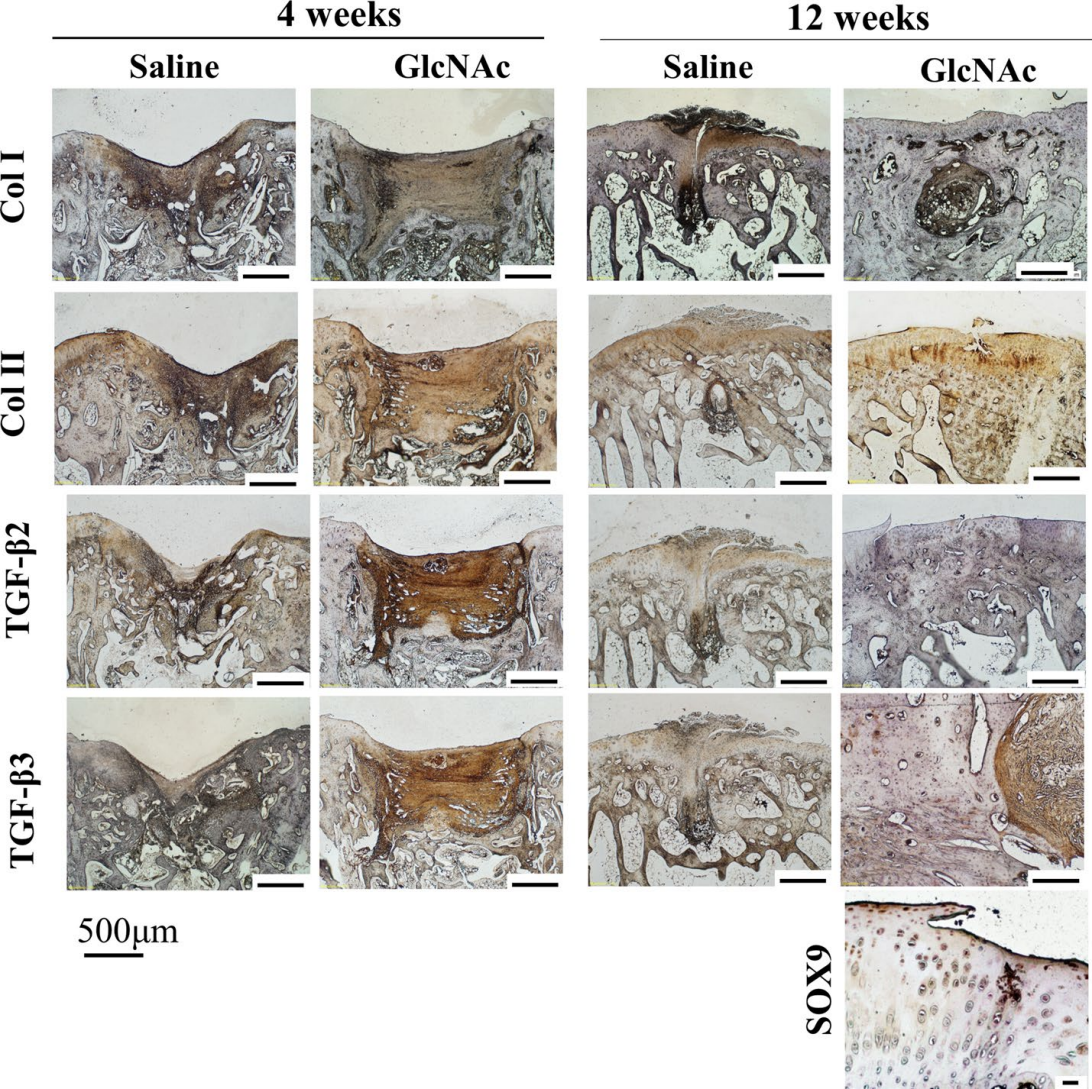
Immunohistochemistry of the reparative areas for the specific proteins COL I and COL II, TGF-β2 and TGF-β3, and SOX9

However, in the GlcNAc group, the migration of undifferentiated chondroblasts together with increasing GAG synthesis had begun in the reparative regions at week 4. Furthermore, the GlcNAc group showed newly generated collagen matrix, coupled with underlying osteoid at the defect edges (Fig. 3a). Moreover, the GlcNAc group displayed higher levels of COL I and COL II than the saline group (Fig. 4). Surprisingly, the two decisive endogenous growth factors (i.e., TGF-β2 and TGF-β3) were highly expressed compared to the saline group (Fig. 4).

#### At 12 weeks after operation

**Fig. 5:**
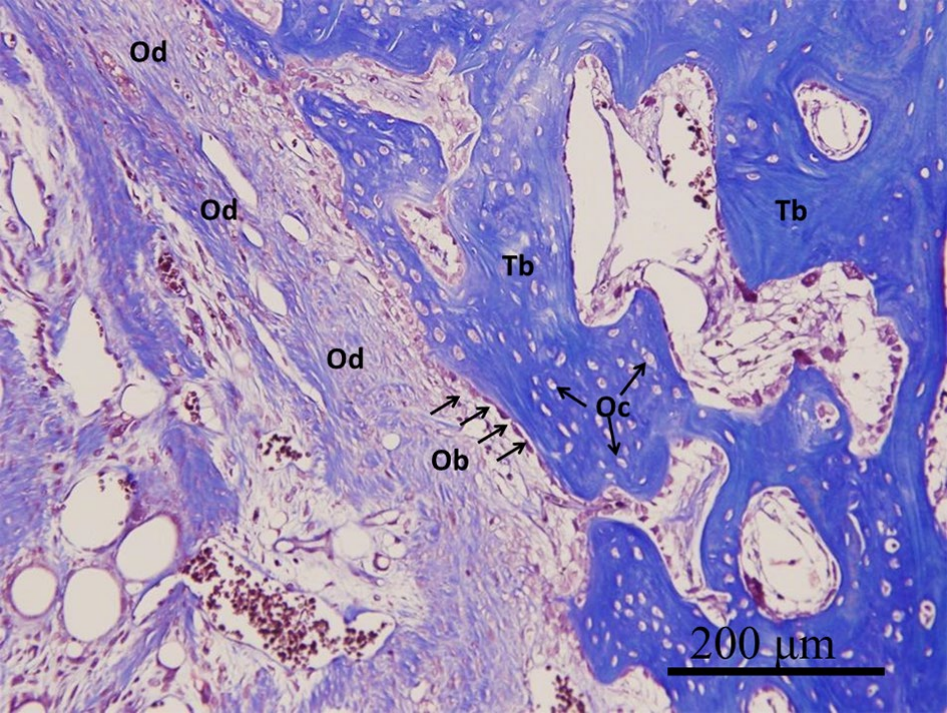
At 12 weeks after operation, trabecular bone (Tb) embedded with mature osteocytes (Oc) in the reparative site was found in the GlcNAc group. Meanwhile, in the healing osteochondral defects, new osteoid matrix (Od) surrounding osteoblasts (Ob), was accompanied by remodeling

Compared to the saline group, the GlcNAc group showed obvious histological changes in both hyaline cartilage regeneration and subchondral bone formation (Fig. 3a). The saline group still showed irregular surfaces, disorganized collagen, and GAG depletion in the adjacent cartilage (Fig. 3a). In contrast, the GlcNAc group showed potential positive outcomes including the regeneration of hyaline-like cartilage corresponding with chondrocytes in lacunae (SOX9+), rich GAG content, reconstructed COL II content (hyaline cartilage) as well as low levels of COL I (fibrocartilage) in the adjacent cartilage and reparative site (Figs. 3a, 5). Furthermore, in the GlcNAc group, the trabecular bone was embedded with osteocytes, along with osteoid matrix surrounding osteoblasts, indicating sound bone remodeling (Fig. 5).

## Discussion

The fate of healing articular cartilage is fundamentally dependent on better healing in the early stages and mechanically subchondral bone regeneration [31]. In this study, the GlcNAc group showed higher levels of tissue calcification, indicating beneficial trends for higher BV/TV and Tb.Th.

The TGF-β superfamily plays significant roles in enhancing chondrocyte growth, chondrocyte proliferation and osteochondrogenic differentiation [25]. TGF-β2 can stimulate chondrocyte proliferation and cartilage regeneration [32, 33]; TGF-β3 may act as a chemotactic molecule (i.e., biological cue) that can recruit bone marrow stem cells, induce the recruited stem cells toward chondrogenesis, and enhance the compressive properties of neocartilage [34]. Based on the current data, we thus suggest that direct manipulation of in situ microenvironments (i.e., GlcNAc injection) provides a better mix of endogenous growth factors and cytokines, especially in the early stage, for cartilage repair. In this study, GlcNAc showed no locally or systemically adverse reactions, consistent with reported outcomes. Previous studies suggest that GlcNAc has an excellent safety profile in humans. In addition, it has been reported that GlcNAc has additional potential advantages over GlcN as a potential therapeutic anti-inflammatory agent. GlcN initiates phosphorylation by glucokinase and competes with glucose for binding to glucokinase in the cell [35], thereby giving rise to GlcN-induced insulin resistance [36]. In contrast, GlcNAc has much lower affinity toward glucokinase than do either glucose or GlcN; thus, it does not respond significantly to glucose metabolism [37].

Based on this study, one possible major reparative mechanism may function through changes to the in situ microenvironments in the reparative site. Transport of nutrients and a subset of signal molecules can be supplied from the synovial membrane to synovial fluid [24]; as an alternative source, nutritional support also diffuses from subchondral bone [38].

## Conclusions

This study demonstrated that intra-articular injection of GlcNAc is safe and has the potential to improve the repair of FTAC in the rabbit knee joint model. Promising outcomes include the improvement of damaged articular surface, formation of hyaline-like cartilage, rich GAG synthesis, and subchondral bone regeneration. This strategy warrants further investigation to support an effective transition from animal models to human patients.

## Abbreviations

HA: hyaluronic acid
GlcN: glucosamine
GlcNAc: N-acetyl-d-glucosamine
PFAA: plasma total free amino acid
FTAC: full-thickness articular cartilage
H&E: hematoxylin and eosin
COL I: collagen type I
COL II: collagen type II
TGF-β: transforming growth factor-β
GAG: glycosaminoglycan
CT: computed tomography
BV/TV: bone volume per tissue volume
Tb.Th: trabecular thickness
